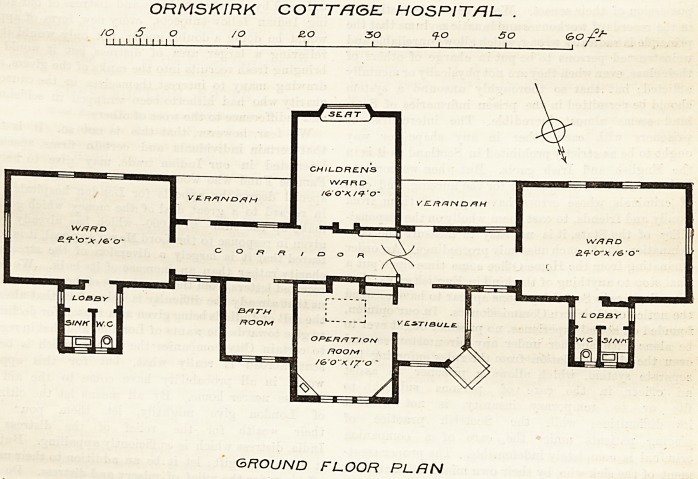# Hospital Construction

**Published:** 1897-01-23

**Authors:** 


### THE INSTITUTIONAL WORKSHOP.

### HOSPITAL CONSTRUCTION.

### ORMSKIRK COTTAGE HOSPITAL.

The accompanying plans represent the nucleus of this building, which at present has no administrative block, the operating-room and children's ward being used for this purpose temporarily. The wards at either end of the corridor are intended to accommodate four beds each, for male and female patients respectively, and have the necessary sanitary appliances connected with them in the usual way by a cross-ventilated lobby. The arrangement of cots proposed in the children's ward is not indicated, nor is the number suggested; but the architect might very well have exercised his discretion as to the position, in all the wards, of the windows, so as to obtain a due amount of light and ventilation between the beds without draught. He scarcely seems to have availed himself of the excellent opportunities which his plan offers for this purpose. It is difficult to conjecture, and therefore impossible to criticise, the proposed arrangement of the administrative blocks; in fact, as the plan stands, it is a puzzle to see where it is going to be fitted in, so as to connect properly with what is already built. The necessity of taking patients of one sex to a bath-room within that portion of the building set apart for the opposite sex is not a good arrangement; and, though the door of the operating-room is a reasonable width for its purpose, the doors into the vestibule out of which the operating-room opens are quite too narrow for practical work. The architect is Mr. C. S. Beeston, of Ormskirk.

ORMSKIRK COTTAGE HOSPITAL.

GROUND FLOOR PLAN

## Figures and Tables

**Figure f1:**